# Aortic Dilatation in Pediatric Patients with Bicuspid Aortic Valve: How the Choice of Nomograms May Change Prevalence

**DOI:** 10.3390/diagnostics13081490

**Published:** 2023-04-20

**Authors:** Gaia Spaziani, Francesca Bonanni, Francesca Girolami, Elena Bennati, Giovanni Battista Calabri, Chiara Di Filippo, Giulio Porcedda, Silvia Passantino, Stefano Nistri, Iacopo Olivotto, Silvia Favilli

**Affiliations:** 1 Pediatric and Transition Cardiology, Meyer Children’s Hospital IRCCS, Viale Pieraccini 24, 50139 Florence, Italy; 2 Department of Experimental and Clinical Medicine, University of Florence, 50100 Firenze, Italy; 3 Cardiology Service, CMSR Veneto Medica, 36077 Altavilla Vicentina, Italy

**Keywords:** bicuspid aortic valve, aortic dilation, aortic nomograms, Q-score

## Abstract

Background: Aortic dilation (AoD) is commonly reported in patients with bicuspid aortic valve (BAV) and has been related to flow abnormalities and genetic predisposition. AoD-related complications are reported to be extremely rare in children. Conversely, an overestimate of AoD related to body size may lead to excess diagnoses and negatively impact quality of life and an active lifestyle. In the present study, we compared the diagnosis performance of the newly introduced Q-score (based on a machine-learning algorithm) versus the traditional Z-score in a large consecutive pediatric cohort with BAV. Materials and methods: Prevalence and progression of AoD were evaluated in 281 pediatric patients ages > 5 and < 18 years at first observation, 249 of whom had isolated BAV and 32 had BAV associated with aortic coarctation (CoA–BAV). An additional group of 24 pediatric patients with isolated CoA was considered. Measurements were made at the level of the aortic annulus, Valsalva sinuses, sinotubular aorta, and proximal ascending aorta. Both Z-scores using traditional nomograms and the new Q-score were calculated at baseline and at followup (mean 4.5 years). Results: A dilation of the proximal ascending aorta was suggested by traditional nomograms (Z-score > 2) in 31.2% of patients with isolated BAV and 18.5% with CoA–BAV at baseline and in 40.7% and 33.3%, respectively, at followup. No significant dilation was found in patients with isolated CoA. Using the new Q-score calculator, ascending aorta dilation was detected in 15.4% of patients with BAV and 18.5% with CoA–BAV at baseline and in 15.8% and 3.7%, respectively, at followup. AoD was significantly related to the presence and degree of aortic stenosis (AS) but not to aortic regurgitation (AR). No AoD-related complications occurred during the followup. Conclusions: Our data confirm the presence of ascending aorta dilation in a consistent subgroup of pediatric patients with isolated BAV, with progression during followup, while AoD was less common when CoA was associated with BAV. A positive correlation was found with the prevalence and degree of AS, but not with AR. Finally, the nomograms used may significantly influence the prevalence of AoD, especially in children, with a possible overestimation by traditional nomograms. This concept requires prospective validation in long-term followup.

## 1. Introduction

Bicuspid aortic valve (BAV) is the most common congenital valve disease [1,2]. The association with a mild aortic dilation (AoD), mostly at the level of proximal ascending aorta, is common, while a minority presents a significant dilation or aortic aneurysm [1,2]. Valve morphology, presence and severity of valve dysfunction, flow abnormalities, and genetic predisposition have been suggested as drivers of dilation [3,4,5,6]. In contrast, associated aortic coarctation (CoA–BAV) has been reported to be inversely related to the presence and degree of AoD.

There is a limited number of reports concerning the prevalence and progression of AoD in the pediatric population and data are not easily comparable because of different demographic and anthropometric characteristics [2,3,7].

Several Z-score calculators have been used to define AoD in children and adolescents, considering sex, height, and weight (or derived measures of body size such as body surface area and body mass index) and eventually age groups [7,8]. However, choosing the type of calculator may be difficult, especially in adolescents with a body size like that of adults. It has been reported that pediatric calculators might fail in the recognition of AoD at extremes in body mass index (overweight or underweight patients) [9].

Although complications related to AoD are rare during pediatric age, the same diagnosis of AoD can have significant effects on the quality of life, especially concerning eligibility for sports activities.

Recently, a new tool for aortic normalcy at the 4levels (aortic annulus, sinus of Valsalva, sinotubular junction, and proximal ascending aorta), the Q-score, has been developed in a large healthy population including > 250 children and adolescents older than 5 years [10]. The Q-score is based on a machine-learning algorithm and diameters do not depend linearly on anthropometric measurements. In addition to the regional Q-score, a “global” Q-score can evaluate the joint distribution of all variables with all 4diameters simultaneously and the whole aortic shape.

In the present study, we aimed to assess the prevalence and progression of AoD in a pediatric cohort of patients, aged 5–18 years, with BAV (isolated or associated with CoA) at the first presentation and after a period of followup, and the potential relationship with valve morphology and associated CoA. We hypothesize that the pediatric Z-scores currently used might overestimate the number of cases with AoD, especially in adolescents with a body surface area (BSA) similar to that of adults. Results obtained by using the Z-score and the new Q-score will be than compared and discussed.

## 2. Materials and Methods

The study cohort consisted of consecutive pediatric patients with isolated BAV or associated with CoA (CoA–BAV), ages > 5 and < 18 years at their first diagnosis, observed in the Cardiology Unit of a regional referral pediatric hospital (Meyer Children’s Hospital, Florence) from 2010 to 2020.

**Figure 1:**
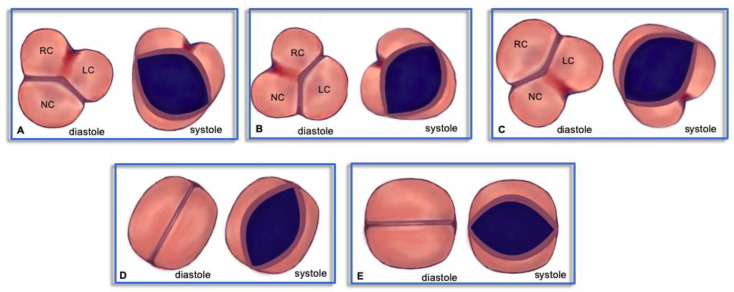
Schematic morphology of bicuspid aortic valve as seen by parasternal short-axis echocardiographic view. RC = right cusp, LC = left cusp, NC = noncoronary cusp. (**A**): fused type with right–left cusp fusion; (**B**): fused type with right noncoronary cusp fusion; (**C**): fused type with left noncoronary cusp fusion; (**D**): 2-sinus type with latero–lateral phenotype; (**E**): 2-sinus type with anteroposterior phenotype.

The morphology of BAV was classified according to recent criteria defined by Michelena et al. [11]: the fused BAV type is characterized by 2 cusps appearing fused with 3 aortic sinuses and frequently there is a raphe; moreover, there are 3 specific phenotypes of fused BAV (right–left cusp fusion, right noncoronary cusp fusion, and left noncoronary cusp fusion, as seen in Figure 1A–C, respectively). The 2-sinus BAV type, presenting with 2 specific phenotypes (latero–lateral and anteroposterior), is less common and results in a 2-sinus/2-cusp type without a raphe (Figure 1D,E).

Patients with BAV and severe aortic stenosis (AS) diagnosed in the first year of life were excluded. Additional exclusion criteria were known genetic syndromes or connective tissue disorders and associated congenital heart disease (except for CoA).

AS and aortic regurgitation (AR) were graded using a 3-level scale of severity (mild, moderate, and severe) based on a multiparametric echocardiographic evaluation. AS was assessed by the determination of peak transvalvular velocity, mean pressure gradient, and valve area. The grading of AR was based on a composite evaluation of the proximal jet width, pressure half-time, holodiastolic flow reversal in the descending aorta, and left ventricle dilatation [12,13].

Two-dimensional (2D) echocardiographic measurements of the aortic root were performed from the parasternal long-axis view at 4 different levels perpendicular of the long axis of aorta: the aortic annulus, measured in end-systole using the inner-to-inner technique at the hinge points of the leaflets and the sinuses of Valsalva, the sinotubular junction, and the ascending aorta, which are measured in end-diastole using the leading-edge to leading-edge technique [14]. A conventional pediatric Z-score and the new Q-score (based on a machine-learning algorithm that uses a vector representing age, sex, BSA, and single or all 4 aortic diameters, thus obtaining a regional Q-score or a global Q-score, respectively) were then applied and compared [8,10]. Since the Q-score is a continuous quantity expressing percentiles, pathological Q-score values were assumed when < 2% [10].

Height (m) and weight (Kg) were measured at the time of the visit, and body surface area (BSA) was calculated by the Du Bois formula as BSA = weight ^0.425^ × height ^0.725^ × 0.007184.

All patients were systematically reassessed with clinical evaluation, electrocardiography, and echocardiography every 6–12 months, depending on the degree of aortic valve dysfunction. The predefined clinical endpoint was a composite of cardiac death, aortic complications (e.g., dissection or rupture), endocarditis, and the need for cardiac surgery or percutaneous procedure on the aortic valve or aortic isthmus. Two different echocardiographic endpoints were considered: (1) progression of AS or AR, defined as an increase by more than 1 grade from baseline to followup evaluation, and (2) significant progressive AoD, defined as development of a Z-score > 2 or a regional Q-score < 2%.

Statistics: Categorical variables were expressed as absolute values and percentage values. Continuous variables were expressed as mean ± standard deviation (SD) or median (interquartile range, IQR) in the case of non-normal distributions. For the continuous variables, the Student *t*-test was used and the Mann–Whitney test depending on the type of distribution. Categorical variables were compared with the χ2 test. *p* < 0.05 was considered statistically significant. Statistical analysis was performed with IBM SPSS for Windows (v. 28.0).

## 3. Results

### 3.1. General Characteristics of the Study Population

The population includes 281 patients aged between 5 and 18 years (mean age 9.18 years) divided into 2 groups: patients with isolated BAV (249) and patients with CoA-associated BAV (32). Additionally, a control group of patients with isolated CoA was considered (24). The general characteristics of the study cohort are described in Table 1.

In the isolated-BAV group, 5 patients underwent intervention on the aortic valve due to severe AS before the first observation: 4 percutaneous valvuloplasties (average 5.75 years) and 1 plastic aortic valve surgery (2 years of age). In the CoA–BAV group, 26 patients underwent previous procedures: 18 end-to-end anastomoses (descending aorta to proximal aortic arch) for CoA, 3 percutaneous treatments of CoA, 2 patched cardiac surgeries, 2 aortic valvuloplasties, and 1 valvular commissurotomy.

In the isolated-CoA group, 11 patients underwent corrective interventions of end-to-end anastomosis and only 1 patient had a percutaneous treatment of the CoA, before the first observation.

The male sex was prevalent in all groups: 77% in the isolated BAV group, 62.5% in the CoA–BAV group, and 75% in the isolated CoA group. The mean age of patients was 9.3 years in the BAV group, 8.3 years in the CoA–BAV group, and 7.8 years in the CoA group.

A positive family history for BAV was reported in 6 patients with isolated BAV (2.4%). Regarding valve morphology, the fused BAV phenotype was present in 83.1% of patients in the isolated BAV group (47.4% with a right–left cusp fusion, 27.3% with a right noncoronary cusp fusion, and 8.4% with a left noncoronary cusp fusion), and in 75% of patients in the CoA–BAV group (43.8% with a right–left cusp fusion, 28.1 % with a right noncoronary cusp fusion, and 3.1% with a left noncoronary cusp fusion). The 2-sinus BAV phenotype was present in 16.9% of patients with isolated BAV and in 25% of patients with CoA–BAV.

In the BAV group, 29 patients (11.6%) had AS: 20 cases were mild and 9 were moderate; in the CoA–BAV group, mild AS was diagnosed in 6 patients (18.7%). No cases of AS were detected in the CoA group.

AR was present in 84 patients (33.7%) with isolated BAV (64 mild cases and 20 moderate cases) and in 6 patients (18.7%) with CoA–BAV (5 mild cases and 1 moderate case); and 3 patients (12.5%) with isolated CoA had mild AR.

The aortic size at 4 levels (median and interquartile range) measured in all groups are reported in Table 2. The aortic diameters were larger in the BAV group compared with the CoA–BAV group and the isolated CoA group.

### 3.2. Clinical Followup

All patients were alive at the end of the followup of 4.5 years (interquartile range 2.35–6.56 years). The mean age at the end of the followup was 13.79 years considering all patient categories.

Forty-four patients were lost at followup; thus, only baseline echocardiographies were available. The overall cohort at followup was represented by 261 patients (221 with isolated BAV, 27 with CoA–BAV, and 13 with isolated CoA). The clinical endpoint occurred in 12/261 (4.6%) cases. In the BAV group, 5 patients underwent procedures on the aortic valve (3 aortic valve replacements, 1 surgical valvulotomy, and 1 percutaneous valvuloplasty). In the CoA–BAV group, 2 patients underwent aortic valve replacement. In the CoA group, 3 patients underwent percutaneous angioplasty treatment with a stent and 2 patients underwent cardiac surgery on aortic isthmus. No aortic dissection or endocarditis occurred in any group.

At the end of followup, AS was present in 27 patients (12.3%) in the BAV group (19 mild cases, 7 moderate cases, and 1 severe case) and in 6 patients (22.2%) in the CoA–BAV group (3 mild cases, 2 moderate cases, and 1 severe case). No cases of AS were present in the group with isolated CoA. AR was still the most frequently associated valve dysfunction with BAV, being present in 116 (52.5%) patients in the BAV group (88 mild, 25 moderate, and 3 severe). In the CoA–BAV group, AR was present in 8 patients (29.6%), 5 mild and 3 moderate. Only 1 case of mild AR was diagnosed in the group of isolated CoA.

### 3.3. Comparison between Different Score Calculators (Z-Score versus Q-Score)

At the end of followup, the percentage of patients with a Z-score > 2 in the BAV group was 7.2%, 5.4%, 9.5%, and 40.7% at the level of the annulus, Valsalva sinus, sinotubular junction, and ascending aorta, respectively. In the CoA–BAV group, patients with dilation (Z-score > 2) of the annulus, Valsalva sinus, sinotubular junction, and ascending aorta were, respectively, 0%, 9%, 11.1%, and 33.3%. In all patients of the isolated CoA group, diameters were < 2 DS at each aortic level.

The Q-score at the level of the annulus, Valsalva sinus, sinotubular junction, and ascending aorta was calculated in 261 patients. In the BAV group, a pathological Q-score was present at baseline in 10%, 7.7%, 6.3%, and 15.4% at the 4 levels (annulus, Valsalva sinus, sinotubular junction, and ascending aorta); and at the end of followup the percentages were 5%, 5.9%, 6.8%, and 15.8%, respectively. In the CoA–BAV group, the Q-scores were 29.6%, 22.2%, 22.2%, and 18.5% at baseline and 0%, 7.4%, 3.7%, and 3.7% at the end of followup. In the isolated CoA group, Q-scores were 30.8%, 23.1%, 7.7%, and 0% at baseline, and at the end of followup, a pathological Q-score was present in 7.7% only at the level of the annulus.

In all BAV patients with AoD, the dilation-interested proximal ascending aorta, no cases of isolated dilation of aortic root (Valsalva sinuses) were observed either at baseline or at the end of followup using either the Z-score or the Q-score.

A comparison between results obtained with Z-score by Gautier nomograms versus regional Q-score is reported in Table 3.

**Figure 2:**
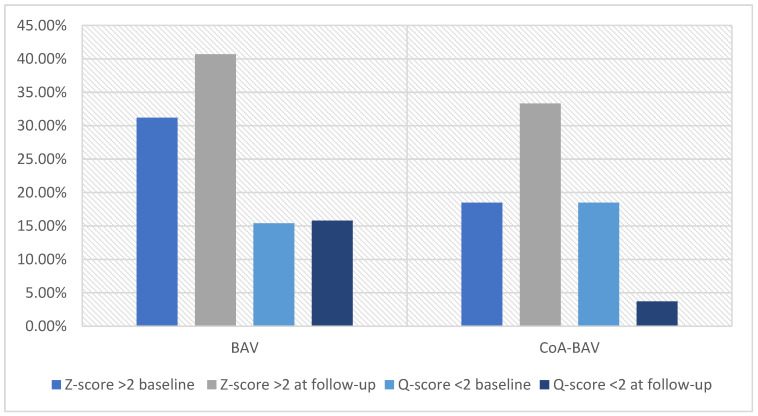
Prevalence of aortic dilation in the 2 groups of patients at baseline and followup; *p* < 0.0001 for both comparisons. BAV = bicuspid aortic valve; CoA = aortic coarctation.

In the BAV group, the proportion of patients with AoD at the level of ascending aorta was significantly different using the Z-score or Q-score (*p* < 0.0001), both at baseline and followup. In the CoA–BAV group, the proportion was similar at the baseline and significantly different at the end of the followup (*p* < 0.0001) (Figure 2).

The global Q-score was also calculated, an additional parameter that considers the 4 aorta diameters, incorporating global morphological information of the whole aorta. The prevalence of AoD by considering the global Q-score is reported in Table 4.

In the BAV group, a Z-score > 2 at the level of the ascending aorta was associated with the presence of AS (*p* < 0.001) and with the degree of stenosis (*p* = 0.009), while no correlation was found with the presence and/or the degree of AR (*p* = 0.06 and *p* = 0.262 respectively). No significant association was found between Z-scores at the other aortic levels and aortic valve dysfunction. In the BAV group, at the end of followup, a pathological Q-score at the level of the ascending aorta was associated with the presence of AS (*p* = 0.004) and with the degree of stenosis (*p* = 0.02), while it was not associated with the presence or the degree of AR. No significant association was found between Q-scores and aortic valve dysfunction at the other aortic levels.

## 4. Discussion

An AoD, mainly at the level of the proximal ascending aorta, has been described in a significant percentage of children with BAV, with progression occurring during the pediatric age [15,16,17,18,19,20,21,22]. Aortic growth in BAV patients appears to be significantly faster when compared with tricuspid valve patients [16,17,18,19,20,21,22]. Ascending AoD was described to progress with increasing age and younger patients presenting with significant dilation are considered at higher risk for complications, including aortic dissection (although exceedingly rare in pediatric age) [22].

In the literature, the presence of dilation has been associated with valve morphology and/or hemodynamic abnormalities due to AS or AR or an abnormal flow pattern; a genetic basis has been proposed [17,20]. Vascular abnormalities (stiffer and less distensible ascending aortas) have been described in BAV versus control children [20,21].

Several studies have reported a relationship between valve morphology and AoD in patients with BAV, with different results [16,17,19,20]. In a large, multicentre retrospective pediatric series [22] a right coronary–left coronary cusp fusion was associated with a larger diameter at aortic sinuses while a right coronary–noncoronary cusp fusion with a larger ascending aorta. In the same population (comprising patients 0–17.9 years old), AR and AS severity were independently associated with dilation of the ascending aorta, while AS severity was related to a smaller diameter of the aortic sinuses. On the contrary, a history of CoA was reported to be inversely related to aortic sinuses and ascending AoD [22,23].

In our population, according to previous reports in pediatric populations [16], no significant dilation of the ascending aorta was found when BAV was associated with CoA. In adults, results are more conflicting [24,25,26].

In our cohort, the presence and degree of AS were associated with AoD in all pediatric ages, while no relationship was found between AR and aortic diameters at any aortic level. The lack of association between severe AS and smaller aortic sinus diameter (which is a typical finding in infantile AS) is probably due to the exclusion from our series of pediatric patients with critical neonatal AS.

The diagnosis of ‘aortic disease’ in pediatric patients with BAV may have important implications in terms of quality of life, primarily related to sports limitations. Although physical activity seems not to drive a greater progression of aortic diameters, at least in a short-term followup [27], the diagnosis of AoD may lead to inappropriate restrictions and disqualification from competitive sport [28].

Moreover, the fear of progression during pediatric age is often of concern for parents and cardiologists, leading to excessively close echocardiographic controls.

Accurate and standardized measurements at different aortic levels are mandatory. However, the nomograms chosen (indexed by sex and BSA) may influence the result, especially for milder degrees of dilation. Namely, referring to a different pediatric population with Marfan Syndrome (MS), significant differences were reported by Rutten et al. [29] when aortic measurements were performed according to different Z-scores. As a threshold of Z-score > 2 represents major criteria for the diagnosis of AoD in MS, the use of different nomograms can have a significant diagnostic impact [29]. Therefore, the definition of “normalcy” and the availability of appropriate tools to assess normalcy is of paramount importance in pediatric age.

In the multicentre study by MIBAVA Consortium, the authors developed a web application to calculate the aortic Z-score, based on the patient’s height, weight, and aortic dimension [22].

Recently, a new tool, independent from anthropometric measurements and based on a machine-learning algorithm (Q-score), has been developed on a large healthy population (over 1,100 patients aged from 5 to 89 years) [10]. The Q-score is based on a different strategy that uses the machine-learning algorithm OC-SVM to identify aortic dimension resulting outside the distribution curve calculated on a large healthy population comprising pediatric patients. The variables used in the algorithm are: age, sex, BSA, and a single aortic diameter obtaining a regional Q-score. When all 4 aortic diameters are included, we obtain a global Q-score. This score allows an analysis of the entire morphology of the aorta and does not depend linearly on anthropometric measurements. To summarize, the ‘philosophy’ of the Q-score, which still deserves to be tested on larger populations with different aortic diseases, is to recognize an abnormal aortic geometry (a ‘nonharmonic’ relationship between aortic diameters), which could be useful to detect a significant AoD, especially where a single aortic diameter is expected to be involved, as is often in BAV.

In our pediatric cohort, an AoD was found in fewer children when the Q-score was used compared to traditional nomograms.

According to previous reports, in our cohort of patients, AoD assessed by both Z-score and Q-score was associated with valve stenosis (and with the degree of the obstruction to the left ventricle outflow).

In all BAV patients with AoD, this involved the proximal ascending aorta, in association or not with other aortic diameters; in no patient was an isolated dilatation of the aortic root detected.

Interestingly, the prevalence of AoD (at the level of ascending aorta) significantly increased during followup when the Z-score was applied (from 30% to 40%) while the prevalence of patients with a pathological Q-score remained unchanged (around 15%). As 40% is significantly higher when compared with previous reports in young adults with BAV [30,31], this suggests a possible overdiagnosis of AoD by conventional methods, especially in adolescents with adult anthropometric data. We speculate that the Q-score might identify the subgroup of children with an associated AoD that deserves a higher level of attention during long-term followup. The exceeding pathological global Q-score in patients with CoA–BAV may be related to a smaller aortic root rather than to AoD, as confirmed by the regional Q-score.

In the overall pediatric population, no major aortic complications occurred during 4.7 years of followup. However the prognostic value of the Q-score should be verified in populations at higher risk for aortic complications (e.g., MS or other connective tissue diseases) and over longer periods.

The main limitations of the present study are represented by the small sample size and the relatively short followup (considering the paucity of expected events during the pediatric age).

Magnetic cardiac resonance could provide additional information about flow abnormalities and might be of use in older pediatric patients in whom it can be easily performed without anesthesia.

## 5. Conclusions

In a large pediatric population with BVA, the high percentage of patients who developed AoD during followup applying the traditional pediatric Z-score was not confirmed when a new score, the Q-score, was applied suggesting a possible over diagnosis using the conventional Z-score, especially in adolescents with adult anthropometric measurements. Further long-term studies are required to assess the evolution of AoD from pediatric age to young adulthood.

## Figures and Tables

**Table 1:** Demographic and echocardiographic characteristics of the study population. BAV = bicuspid aortic valve; CoA = aortic coarctation; BSA = body surface area; R–L = right–left; R-N = right noncoronary; L-N = left noncoronary.

VARIABLES	BAV	CoA–BAV	CoA
Male	192 (77.11%)	20 (62.50%)	18 (75%)
Age (years)	9.3 (4.5–17.9)	8.3 (5–16.9)	7.8 (5–16)
BSA (m^2^)	1.35 +/− 0.81	1.16 +/− 0.55	1.12 +/− 0.49
BAV morphology			
2-sinus type	42 (16.9%)	8 (25%)	0
R–L cusp fusion	118 (47.4%)	14 (43.8%)	0
R-N cusp fusion	68 (27.3%)	9 (28.1%)	0
L-N cusp fusion	21 (8.4%)	1(3.1%)	0
Aortic Stenosis (N)	29 (11.6%)	6 (18.7%)	0
Mild	20 (8%)	6 (18.7%)	0
Moderate	9 (3.6%)	0	0
Severe	0	0	0
Aortic regurgitation (N)	84 (33.7%)	6 (18.7%)	3 (12.5%)
Mild	64 (27.5%)	5 (15.6%)	3
Moderate	20 (8%)	1 (3.1%)	0
Severe	0	0	0

**Table 2:** Aortic size at annulus, sinus of Valsalva, sinotubular junction, and proximal ascending aorta level (median diameters and IQR). IQR = interquartile range; BAV = bicuspid aortic valve; CoA = aortic coarctation.

Aortic Diameters	BAV [Median and IQR]	CoA–BAV [Median and IQR]	CoA [Median and IQR]
Anulus (mm)	17 (10–30)	15 (10–23)	14 (10–20)
Valsalva Sinus (mm)	22.5 (14–38)	20.5 (11–30)	18 (15–27)
Sinotubular junction (mm)	19 (12–37)	17 (10–25)	15 (12–23)
Ascending aorta (mm)	22 (12–41)	19 (12–37)	16.5 (14–25)

**Table 3:** Prevalence of aortic dilation by comparing Z-scores and Q-scores. BAV = bicuspid aortic valve; CoA = aortic coarctation; STJ = sinotubular junction.

Baseline	BAV Z-Score > 2	BAV Q-Score < 2%	CoA–BAV Z-Score > 2	CoA–BAV Q-Score < 2%	CoA Z-Score > 2	CoA Q-Score < 2%
Anulus	5.4%	10.0%	0%	29.6%	0%	30.8%
Valsalva Sinus	3.2%	7.7%	0%	22.2%	0%	23.1%
STJ	7.2%	6.3%	7.4%	22.2%	0%	7.7%
Ascending Aorta	31.2%	15.4%	18.5%	18.5%	0%	0%
**At Followup**	**BAV** **Z-Score > 2**	**BAV** **Q-Score < 2%**	**CoA–BAV** **Z-Score > 2**	**CoA–BAV** **Q-Score < 2%**	**CoA** **Z-Score > 2**	**CoA** **Q-Score < 2%**
Anulus	7.2%	5.0%	0%	0%	0%	7.7%
Valsalva Sinus	5.4%	5.9%	9%	7.4%	0%	0%
STJ	9.5%	6.8%	11.1%	3.7%	0%	0%
Ascending Aorta	40.7%	15.8%	33.3%	3.7%	0%	0%

**Table 4:** Prevalence of aortic dilation considering global Q-score at baseline and followup. BAV = bicuspid aortic valve; CoA = aortic coarctation.

Global Q-Score < 2%	BAV	CoA–BAV	CoA
At Baseline	12 (5.4%)	6 (22.2%)	0 (0%)
At Followup	20 (9%)	2 (7.4%)	0 (0%)

## Data Availability

Data are contained within the article.

## References

[1] Ward R.M., Marsh J.M., Gossett J.M., Rettiganti M.R., Collins R.T. (2018). Impact of bicuspid valve morphology on aortic valve disease and aortic dilation in pediatric patients. Pediatr. Cardiol..

[2] Spaziani G., Girolami F., Arcieri L., Calabri G.B., Porcedda G., Di Filippo C., Surace F.C., Pozzi M., Favilli S. (2022). Bicuspid aortic valve in children and adolescents: A comprehensive review. Diagnostics.

[3] Warren A.E., Boyd M.L., O’Connell C., Dodds L. (2006). Dilatation of the ascending aorta in pediatric patients with bicuspid aortic valve: Frequency, rate of progression and risk factors. Heart.

[4] Allen B.D., Van Ooij P., Barker A.J., Carr M., Gabbour M., Schnell S., Jarvis K.B., Carr J.C., Markl M., Rigsby C. (2015). Thoracic aorta 3D hemodynamics in pediatric and young adult patients with bicuspid aortic valve. J. Magn. Reson. Imaging.

[5] Bissell M.M., Hess A.T., Biasiolli L., Glaze S.J., Loudon M., Pitcher A., Davis A., Prendergast B., Markl M., Barker A.J. (2020). Aortic dilation in bicuspid aortic valve disease: Flow pattern is a major contributor and differs differs with valve fusion type. Circ. Cardiovasc. Imaging.

[6] Pepe G., Nistri S., Giusti B., Sticchi E., Attanasio M., Porciani C., Abbate R., Bonow R.O., Yacoub M., Gensini G.F. (2014). Identification of fibrillin 1 gene mutations in patients with bicuspid aortic valve (BAV) without Marfan syndrome. BMC Med. Genet..

[7] Siurana J.M., Sabaté-Rotés A., Ayerza A., Jimenez L., Figueras-Coll M., Gonzalez M.A., Jiménez L., González A., Escribà S., Collell R. (2021). Adolescents with bicuspid aortic valve: Which criteria should we use for aortic dilatation?. Int. J. Cardiol..

[8] Gautier M., Detaint D., Fermanian C., Aegerter P., Delorme G., Arnoult F., Milleron O., Raoux F., Stheneur C., Boileau C. (2010). Nomograms for aortic root diameters in children using two-dimensional echocardiography. Am. J. Cardiol..

[9] Braley K.T., Tang X., Makil E.S., Borroughs-Ray D., Collins R.T. (2017). The impact of body weight on the diagnosis of aortic dilation-misdiagnosis in overweight and underweight groups. Echocardiography.

[10] Frasconi P., Baracchi D., Giusti B., Kura A., Spaziani G., Cherubini A., Favilli S., Di Lenarda A., Pepe G., Nistri S. (2021). Two-Dimensional Aortic Size Normalcy: A Novelty Detection Approach. Diagnostics.

[11] Michelena H.I., Della Corte A., Evangelista A., Maleszewski J.J., Enriquez-Sarano M., Bax J.J., Otto C.M., Schäfers H.J. (2020). Speaking a common language: Introduction to a standard terminology for the bicuspid aortic valve and its aortopathy. Prog. Cardiovasc. Dis..

[12] Baumgartner H., Hung J., Bermejo J., Chambers J.B., Evangelista A., Griffin B.P., Iung B., Otto C.M., Pellikka P.A., Quiñones M. (2009). Echocardiographic assessment of valve stenosis: American Society of Echocardiography/European Association of Echocardiography recommendations for clinical practice. J. Am. Soc. Echocardiogr..

[13] Lancellotti P., Tribouilloy C., Hagendorff A., Moura L., Popescu B.A., Agricola E., Monin J.L., Pierard L.A., Badano L., Zamorano J.L. (2010). European Association of Echocardiography recommendations for the assessment of valvular regurgitation: Part 1. Aortic and pulmonary regurgitation (native valve disease). Eur. J. Echocardiogr..

[14] Lang R.M., Bierig M., Devereux R.B., Flachskampf F.A., Foster E., Pellikka P.A., Picard M.H., Roman M.J., Seward J., Shanewise J.S. (2005). Recommendations for chamber quantification: A report from the American Society of Echocardiography’s Guidelines and Standards Committee and the Chamber Quantification Writing Group, developed in conjunction with the European Association of Echocardiography, a branch of the European Society of Cardiology. J. Am. Soc. Echocardiogr..

[15] Fatehi Hassanabad A., King M.A., Di Martino E., Fedak P.W.M., Garcia J. (2022). Clinical implications of the biomechanics of bicuspid aortic valve and bicuspid aortopathy. Front. Cardiovasc. Med..

[16] Blais S., Meloche-Dumas L., Fournier A., Dallaire F., Dahdah N. (2020). Long-term risk factors for dilatation of the proximal aorta in a large cohort of children with bicuspid aortic valve. Circ. Cardiovasc. Imaging.

[17] Fernandes S., Khairy P., Graham D.A., Colan S.D., Galvin T.C., Sanders S.P., Singh M.N., Bhatt A., Lacro R.V. (2012). Bicuspid aortic valve and associated aortic dilation in the young. Heart.

[18] Merkx R., Duijnhouwer A.L., Vink E., Roos-Hesselink J.W., Schokking M. (2017). Aortic Diameter Growth in Children With a Bicuspid Aortic Valve. Am. J. Cardiol..

[19] Mahle W.T., Sutherland J.L., Frias P.A. (2010). Outcome of isolated bicuspid aortic valve in childhood. J. Pediatr..

[20] Rumman R.K., Slorach C., Hui W., Lopez C., Larios G., Fan S., Franco-Cereceda A., Loeys B., Mohamed S.A., Dietz H. (2022). Regional vascular changes and aortic dilatation in pediatric patients with bicuspid aortic valve. Can. J. Cardiol..

[21] Bradley T.J. (2022). Does routine measurement of aortic stiffness in children with bicuspid aortic valve rovide an opportunity to better personalize care?. Can. J. Cardiol..

[22] Grattan M., Prince A., Rumman R.K., Morgan C., Petrovic M., Hauck A., Young L., Franco-Cereceda A., Loeys B., Mohamed S.A. (2020). Predictors of Bicuspid Aortic Valve-Associated Aortopathy in Childhood: A report from the MIBAVA Consortium. Circ. Cardiovasc. Imaging.

[23] Sophocleous F., Biffi B., Milano E.G., Bruse J., Caputo M., Rajakaruna C., Schievano S., Emanueli C., Bucciarelli-Ducci C., Biglino G. (2018). Aortic morphological variability in patients with bicuspid aortic valve and aortic coarctation. Eur. J. Cardio-Thorac. Surg..

[24] Oliver J.M., Alonso-Gonzalez R., Gonzalez A.E., Gallego P., Sanchez-Recalde A., Cuesta E., Aroca A., Lopez-Sendon J.L. (2009). Risk of Aortic Root or Ascending Aorta Complications in Patients With Bicuspid Aortic Valve With and Without Coarctation of the Aorta. Am. J. Cardiol..

[25] Michelena H.I., Khanna A.D., Mahoney D., Margaryan E., Topilsky Y., Suri R.M., Eidem B., Edwards W.D., Sundt T.M., Enriquez-Sarano M. (2011). Incidence of Aortic Complications in Patients With Bicuspid Aortic Valves. JAMA.

[26] Duijnhouwer A., van den Hoven A., Merkx R., Schokking M., van Kimmenade R., Kempers M., van Dijk A., de Boer M.J., Roos-Hesselink J. (2020). Differences in Aortopathy in patients with a Bicuspid Aortic Valve with or without Aortic Coarctation. J. Clin. Med..

[27] Monda E., Fusco A., Della Corte A., Caiazza M., Cirillo A., Gragnano F., Giugliano M.P., Citro R., Rubino M., Esposito A. (2021). Impact of Regular Physical Activity on Aortic Diameter Progression in Pediatric Patients with Bicuspid Aortic Valve. Pediatr. Cardiol..

[28] D’Ascenzi F., Valentini F., Anselmi F., Cavigli L., Bandera F., Benfari G., D’Andrea A., Di Salvo G., Esposito R., Evola V. (2021). Working group of echocardiography of the Italian Society of Cardiology. Bicuspid aortic valve and sports: From the echocardiographic evaluation to the eligibility for sports competition. Scand. J. Med. Sci. Sport..

[29] Rutten D.W., Aarts-Janssen I.J., Kempers M.J., Reimer A.G., Ten Cate F.E.U., Loeys B.L., Slieker M.G. (2021). Comparability of different Z-score equations for aortic root dimensions in children with Marfan syndrome. Cardiol. Young.

[30] Michelena H.I., Della Corte A., Prakash S.K., Milewicz D.M., Evangelista A., Enriquez-Sarano M. (2015). Bicuspid aortic valve aortopathy in adults: Incidence, etiology, and clinical significance. Int. J. Cardiol..

[31] Nistri S., Basso C., Marzari C., Mormino P., Thiene G. (2005). Frequency of bicuspid aortic valve in young male conscripts by echocardiogram. Am. J. Cardiol..

